# Georgia State Medical Association

**Published:** 1894-05

**Authors:** 


### Society Reports.

### GEORGIA STATE MEDICAL ASSOCIATION.

*Abstract of the Proceedings of the Forty-fifth Annual Meeting, Held in Atlanta, April 18, 19 and 20, 1894.*

### FIRST DAY—MORNING SESSION.

The Association convened in Concordia Hall, and was called to order by the President, Dr. W. H. Elliott, of Savannah, at 10 A. M.

Prayer was offered by the Rev. I. S. Hopkins.

An address of welcome was delivered by the Hon. N. J. Hammond, of Atlanta, and President Elliott responded thereto in behalf of the Association.

Dr. J. B. S. Holmes, of Atlanta, took the chair, and President Elliott delivered his address. He selected for his subject "The Duty of the Physician to his Patient." He began by refreshing the memory of the members by not only a recital of the law, but asked attention to some practical applications of its precepts. He quoted largely from the American and English Encyclopedia of Law, and pointed out the obligations the law implies upon the physician. Directing attention to the code of medical ethics of the American Medical Association, he said that this code was formally adopted by the Constitution of the Medical Association of Georgia, and the fact of its membership made it binding upon the members. It was an obligation voluntarily assumed, and therefore they were bound by the highest dictates of honor to faithfully observe its injunctions. He closed by saying that the standard of responsibility was high, but that it should only increase our zeal to attain it. "If we regard our patients as men and brethren, we will, without the restraint of law and code, follow the Golden Rule, and 'do unto others as we would have them do unto us.' If we are faithful to this, what is our reward? Not only 'the promise of the life that now is,' but when the Son of Man, who was our brother here on earth, shall be the King upon the Throne of his Glory, he will say, 'Inasmuch as ye have done it unto one of the least of these my brethren, ye have done it unto Me.' "

The address elicited considerable applause throughout its delivery.

The report of the Committee of Arrangements was then read by the Chairman, Dr. Willis F. Westmoreland, of Atlanta.

Dr. C. D. Hurt, of Atlanta, followed with the report of the Committee on Program.

The reading of papers was then proceeded with, and the first paper read was by Dr. W. C. Humphries, of Acworth, entitled, "The Treatment of Pneumonia," in which he confined himself mostly to the acute lobar variety. The majority of cases with which he comes in contact assume from the beginning the sthenic type and require active or depleting measures. If called to treat the patient in the first stage of the disease he resorts to moderately active catharsis and an agent that will arouse the dormant secretions and assist nature to eliminate from the system the effete products produced by the disease. For this purpose he finds no better remedy than calomel, and usually prescribes from six to ten grains to an adult in combination with Dover's powders or some preparation of opium and bismuth subnitrate. His next effort is directed to reducing the force and frequency of the heart's action, and for this purpose he has tried several remedies, such as aconite, gelsemium, the coal tar derivatives, etc., but none of them have given so great satisfaction or such uniform results in his hands as veratrum viride. He uses this oftener than any other heart sedative in this disease and it seldom ever fails him. He does not give it to such an extent as to produce nausea, but in small and often repeated doses. With veratrum he combines turpentine in the form of an emulsion. The author then dwelt upon external applications in the treatment of the disease.

Dr. O. H. Buford, of Cartersville, followed with a paper on the "Fluid Extract of Jaborandi as an Abortive Treatment in Pneumonia." The author thinks that in this drug we have a remedy that in many cases will force a crisis when given early in the congestive stage. He had used it for several years with good effect. After giving fluid extract of jaborandi, he follows it with quinine and stimulants if indicated. The author then reported three cases in which he used this treatment.

Dr. T. M. Geenwood, of Mineral Bluff, read a paper on the "Abortive Treatment of Pneumonia." He said his mode of treating the disease was to apply a mustard plaster over the entire portion of the lung affected. He usually makes or has them made under his special directions, using the purest brand mixed with water only, letting the plaster remain on the patient for six hours. He next investigates the condition of the bowels, the secretions in general, and, if necessary, gives a mercurial. If this does not cause a free evacuation of the bowels, he administers Epsom salts or castor oil. He next prepares a solution of salycilate of soda, containing about sixty grains of the soda to an ounce of water, giving one teaspoonful every two or three hours, according to the intensity ot the pain and the amount of fever. Expectorants in his hands have done little or no good, generally doing harm, the great objection to this treatment being, when long continued, its rejection by the stomach. He considered this treatment abortive, for in his hands it has either been abortive or curative. A trial of over fifty cases have given varying success, without a single death.

Dr. C. S. Webb, of Atlanta, called attention to the fluid extract of ergot to reduce the temperature in the first stage of pneumonia. Ergot is to some extent a heart depressant. It slows the heart and reduces the temperature, and in his opinion is not so dangerous a drug as the fluid extract of jaborandi.

Dr. R. R. Kime, of Atlanta, said it was absolutely essential in the treatment of pneumonia to distinguish between the first stage of the affection and that of absorption of the circulation after consolidation had taken place and adapt the treatment to these conditions, otherwise failures would be made. Bleeding and the use of the coal tar derivatives should have a limited place in the treatment of the disease. It was worse than folly to tax the heart to its utmost capacity by the use of cardiac remedies. But the physician should increase the power of the heart, equalize the circulation in order to get rid of the material in the lung. At this stage many cases could be tided over the critical period by the hypodermatic use of strychnine.

Dr. W. H. Doughty, of Augusta, formulated the treatment of the disease under four heads: (1) Give the patient plenty of fresh air. (2) Keep him clean. (3) Feed him, and (4) come just as near as possible to letting him alone.

Dr. Wm. O'Daniel, of Bullard, considered the disease self-limiting and advocated supportive treatment. The patient needs to be supported early, that if the physician waited for any length of time the loss could not be made up.

### AFTERNOON SESSION.

Dr. H. J. Williams, of Macon, read a paper entitled "A Case of Sarcoma of the Ilium Following a Railway Injury." The patient was 32 years of age, strong and healthy, a trainmaster by occupation. There was a wreck on his division, and it was important that the roadway should be rapidly cleared, and so he went to the scene of the accident. A guide rope was attached by one end to a locomotive and by the other to some part of the wreck, so that the power of the locomotive could be used in removing the obstacle. The The patient was standing by this rope; it broke under the strain, he became entangled and was dragged some distance by the engine, striking his right buttock against the corner of a cross-tie. He felt no inconvenience from the accident, except a momentary pain following a contusion near the exit of the great sciatic nerve, and returned to work. A month later he consulted Dr. Williams for a pain in the right buttock. One day while using deep injections the author thought he felt the end of his needle scraping denuded bone. This he mentioned to the patient and then received the first intimation of the injury received at the wreck. From this he constructed a theory that the bone had been injured, and, denuded of periostium, was pressing upon the sciatic nerve at the notch in the pelvis. The doctor suggested the possibility of an operation, which at this time was declined. About six weeks or two months later he operated. Beginning his incision as for aneurism of the sciatic artery, at the posterior superior spine of the ilium, it was carried over the top of the larger tumor of the greater trochanter, and the tissues were dissected down to the tumor. It was situated on the flange of the ilium just above the exit of the sciatic nerve and attached to the bone, though unattached to the muscles over it. The upper and posterior third of the great sciatic notch was denuded of periostium and a stellate fracture radiating from the notch upward and backward for an inch and one-half was found, the bone being pressed slightly inward upon the sciatic nerve. In attempting to cut out the tumor a cavity in it was opened followed by a gush of fluid, and a small clot. He feared he had opened an aneurismal sac. Running his finger into this sac to find the opening of the artery, he found the interior of the cavity encrusted with small spiculæ of bone. There was no collapsing of the cavity nor any further escape of fluid after the first gush from it. He removed several small pieces of denuded bone and as much as possible of the tumor. Not a drop of pus was found in the growth. The operation was a very bloody one and the patient was very much depressed. The wound was packed with iodoform gauze and dressed antiseptically, the patient put between blankets, stimulants administered hypodermatically and hot bottles used to bring on reaction. Examination of the growth disclosed it to be a subperiosteal sarcoma of the mixed type. The patient did well for weeks following the operation, but died some forty-three days after the operation and nearly eight months after the accident.

Dr. J. McFadden Gaston, of Atlanta, read a paper entitled, "Operation for Fibro-Cystic Sarcoma Involving the Right Inferior Maxillary Bone." The patient, a colored woman, was thirty-five years of age, and presented herself with a reproduction of the growth at the clinic of the Southern Medical College. Examination revealed a large mass involving the region from the angle to the mental curve of the right lower maxillary bone, extending upward to the zygomatic arch and inward to the root of the tongue. There was a protuberance of indurated substance in the middle and a tense elastic portion above, with a larger formation of the same character below. The entire cavity of the mouth on the right side was filled with the tumor. The previous operation was performed about two years ago, and the patient states that the growth commenced some six years prior to that time. An operation being advised and consented to, the patient was instructed to take two compound cathartic pills night and morning to procure free evacuation from the bowels, and to refrain from food, on the morning preparatory to undergoing the operation. At the outset of the operation a strong silk ligature was passed through the tongue and its ends knotted together so as to form a loop, which was to be made available to prevent the tongue from dropping back and interfering with respiration at a subsequent stage of the operation. This precaution was made requisite from the record of cases in which suffocation has resulted from its ommission when the lateral attachments of the tongue have been severed. The next step was the extraction of two of the lower incisor teeth, where it was expected to divide the thick bony structure subsequently, either with the saw or bone forceps. This would effect its detachment in front and another section behind the angle of the jaw, though the condyloid neck would separate the bone behind, so that all the intervening portion of the lower maxillary bone on the right side should be detached with the tumor.

Dr. Gaston's greatest concern in regard to hemorrhage connected with the operation was relieved by the expectation of leaving the condyloid process and thus obviating the risk of wounding the internal maxillary artery by its removal. Having planned to accomplish the incision and dissection, so far as possible, without entering the buccal cavity, and thus avoid the entrance of blood into fauces, he first divided the skin from a point immediately below the middle of the lower lip along the line of cicatrix from the former incision back to the angle of the right lower jaw, and thence upward to the prominence of the zygoma, keeping away from the temporal artery in front of the ear. The skin was then dissected back on the lower side from the tumor and in like manner above to the zyyomatic arch, thus exposing the exterior surface of the diseased structure without entering the buccal cavity. The few cutaneous arteries which were divided were secured with artery forceps in the course of the dissection on the lower border. Dr. Gaston outlined the further details connected with the operation. Following the operation there was a steady increase of coma, with a gradual decline of vital force until the death of the patient six days and four hours after the operation. With the favorable state of the wound in the progress of this case, and the final retention of urine, Dr. Gaston is disposed to consider the coma of uræmic origin, but has not sufficient data upon which to base this opinion. The cause of death was given as septicæmia in his certificate.

"Malignant Growths of the Skin: Their Diagnosis and Treatment." This was the title of a paper read by Dr. M. B. Hutchins, of Atlanta, in which he said that while not all the growths to be described are strictly malignant, in a literal sense, the term applies in that they are either malignant or terminate in malignancy. We have the carcinomata of the skin with epithelioma as a related disease, and the sarcomata. Epithelioma being more frequently met with, Dr. Hutchins described this first. He said that authors described three varieties of epithelioma, the superficial, the deep-seated, the papillary. These three varieties were then interestingly described.

Coming to the treatment of epithelioma, he said this must be limited to local measures, as internal treatment has no value. Incomplete destruction or removal is liable to do more harm than good. Whether caustics, the actual cautery, the galvano-cautery, ecraseur or knife are used, the operation must be thorough and the destruction of the growth complete. The means used must be governed by the size, extent and situation of the growth. The caustics are said to be best for superficial growths. Caustic potash may be bored into the diseased tissues, and into its border, any excess being neutralized by dilute acetic acid. It is not an exceedingly painful method. Pure carbolic acid, followed in a few minutes by nitrate acid is mentioned by Shoemaker. Chloride of zinc has been used, but it is very painful and has no advantages. Marsden's paste was referred to as an excellent application for certain forms of epithelioma, as the small, not too deep. The curette may be used for very superficial growths or preceding the caustic application.

Regarding the use of the knife, the author said there was a popular prejudice against it, and people would submit to anything in preference to its employment. There are recurrence after the knife, but their frequency, their malignancy, and their terribly destructive action following inefficient or incompetently used applications remove none of the fear of the knife. For the majority of cases the knife is the only remedy, and it is to be preferred where not mechanically contra-indicated. The deep-seated and the papular forms especially require excision. Every visible particle of the disease must be removed, and with it a perfect coating of healthy tissue.

There is little to be said regarding the treatment of the cutaneous carcinomata and sarcomata. The carcinoma is so likely to be secondary that the local treatment is of doubtful use. We must direct our attention to general palliative and supportive measures.

Dr. Arthur G. Hobbs, of Atlanta, read a paper entitled, "Treatment of Corneal Ulcers with the Galvano-Cautery."

Dr. J. W. Hallum, of Carrollton, contributed a paper on, "Chronic Sore Legs and How to Cure Them," in which he said that chronic sore legs were the result of a disease that we often meet in our healing journey. In the remarks of his paper he included specific and maligant ulcers, and discussed a simply chronic ulcer that has refused to heal from all efforts of nature that have been brought to bear upon it. The author had been curing such ulcers by painting them with carbonate of lead and linseed oil, in the following proportion:

Pure white lead (ground in oil)............................................℥ xv.

Raw linseed oil,..................................................................℥ vi.

Mix well and sign, paint the ulcer once or twice a day after washing it with warm water. Dry well before painting. The best thing to apply the remedy with is a camel's hair brush. He was not able to tell how this application effects a cure; but carbonate of lead is sedative, astringent, and probably possesses disinfectant powers all of which the author considers quite essential in the cure of these ulcers. The shortest time he has been able to accomplish a cure by this method was six day. The ulcer was two and one half inches in a diameter and of three years standing, but had not penetrated the entire true skin.

SECOND DAY—MORNING SESSION.

Dr. H. Perdue, of Barnesville, read a paper on "Diagnosis." He said the ability to make an early and quick diagnosis should be sought, and, if possible, attained by every physician. It enables one oftentimes to abort diseases, and even when this cannot be done the violence of the attack may often be modified, and its duration abridged. Thoroughness should be the aim of every physician. None should be satisfied with attainments short of the ability to make a diagnosis through scientific and practical methods of research.

Dr. Luthur B. Grandy, of Atlanta, read a paper entitled " Suture of the Tendo Achillis," in which he reported a case and exibited the patient.

Similar cases were reported by Drs. Batty, of Rome, and Wm. O'Daniel. of Bullard.

Dr. J. B. S. Holmes then read a paper entitled, "Making and Closing the Incision in Abdominal Surgery." He said that many surgeons, in doing abdominal work, pay more attention to time than the details that are necessary to secure results that are free from sequelæ. This not only reflects seriously upon the surgeon, but leaves the patient in many instances a sufferer for life, requiring often a second operation to cure what might have been prevented. A smooth wound with no cicatricial tissue, no abscess scars, and no ventral hernia, is a pleasing sight, not only to the surgeon but to the patient. He would not pretend to say that the sequelæ following abdominal operations can always be prevented, but certainly with proper care and attention to the technique of the operation, if not prevented entirely, they can be reduced to a minimum. The author then went into the details of closing the incision in abdominal operations.

Dr. F. W. McRae, of Atlanta, contributed a paper on, " Appendicitis with Report of Cases."

The author presented some tentative conclusions and opinions culled from papers and discussions which had fallen under his notice within the last few months.

In the fulminant cases there was no question as to the proper treatment. Nothing but an early operation would save the patient; but in the milder cases there was much diversity of opinion as to how we should proceed. The author's individual experience was confined to six cases of well defined appendicitis. He had seen several other cases where there were some indications of inflammatory trouble in the right iliac fossa, but no well-defined lesion. They all got well. Of the six cases of appendicitis, one, a child of two years, died before he saw the case, after an illness of five days. From the history of the case and a careful examination of the body he had no doubt as to the diagnosis. Of the other five cases, two passed through one attack, one through two attacks, and one through four attacks of well-marked appendicitis in his hands. In all, except one, he advised operation. All declined it. All except one are now in good health. The one who has had four attacks, he thinks, will require an operation later, as there is a well-defined persistent tumor in the right iliac fossa.

Dr. Samuel C. Benedict, of Athens, delivered the "Orator's Address." He selected for his subject "Suggestion and Its Therapeutic Uses." By suggestion is meant simply an impression made from without, through one of the five senses, upon the cells of the cerebral cortex; it is a sensory impression by which either motor phenomena only are developed, or mental action such as revival of memory. In some instances a language used in childhood and long since forgotten, has been brought again into speech as clearly as though never lost. Auto-suggestion arises without external impressions and is typical in dreams or delirium. Now, as explanatory of such phenomena, a working hypothesis has been formulated by Hudson which explains better than any previous theory the causes for such phenomena. His reasoning is that man has two minds, one of which he calls the objective, and one the subjective mind, and further, that the subjective mind, which is the storehouse of the memory and the seat of the emotions, is constantly amenable to control by suggestion, and is incapable, of inductive reasoning.

Dr. P. R. Cortelyou, of Marietta, reported an interesting case of inflammation of external auditory canal following facial erysipelas, in a woman sixty-eight years of age.

Dr. Dunbar Roy, of Atlanta, read a paper on "Chronic Otorrhea, Its Significance and Treatment." This paper dealt with, (1) Chronic discharges from the external auditory meatus; (2) polypi and foreign bodies, and (3) discharges originating from and dependent upon pathological conditions of the middle ear or its contiguous cavities. With reference to the treatment, the author said that painstaking research into the history of every case ; the correlation and comparison of all phenomena ; a thorough knowledge of the normal and abnormal appearances of the parts, coupled with a sufficient amount of clinical experience, were necessary for the treatment of all ear diseases.

Dr. J. H. Shorter, of Macon, read a paper entitled "Treatment of Cataract." He said all operations for removal of cataract have been by one of three methods: Depression, discission, or extraction. These three methods were then described in detail, and the indications for each method pointed out.

Dr. H. McHatton, of Macon, followed with a paper on "Teething." See page 233.

Dr. Richard Douglas, of Nashville, addressed the Association by invitation on "Surgical Shock."

Dr. R. H. Taylor, of Griffin, read a paper on "An Operation for Hemorrhoids." He describes the operation as follows : After preparing the patient for operation in the usual way, fix the hemorrhoid with vulsellum forceps or a stout tenaculum, which is firmly held by an assistant. Instead of cutting a groove around the base of the pile for your ligature in the usual way, make an eliptical incision through the mucous membrane, including the hemorrhoid in the ellipsis. Turn back a small flap of mucous membrane from the base of the tumor. Now have the assistant make firm traction on the pile while you pass a double ligature of stout chromacized catgut through the pedicle, which may be much reduced in size in some instances by separating it from the muscular coat of the bowel from below upward. Firmly secure the ligature and cut away the tumor as close to the ligature as possible, only leaving enough stump to safely prevent slipping of the ligature. Then close the flaps over the ligated stump with a suture of the chromacized catgut. This leaves you with a closed wound which, if aseptic, will practically heal in four days.

Dr. R. P. Cox, of Rome, read a paper entitled, "Sacrificial Surgery of the Ovaries, Tubes and Uterus for Uterine Fibroids and Uterine Cancer." In the treatment of uterine fibroids and uterine cancer, radical interference is often the best hope for the one and always the only hope for the other. The rather indefinite, though usually given, moral ground of justification for excision of the pelvic organs is to save life, to preserve from destruction a patient's usefulness or her capacity for the rational pursuit of happiness, or to relieve serious and permanent impairment of these natural rights. The author said that any medical man who advises or performs these radical operations on vague indication, without the ability to give an honest and reasonably intelligent opinion as to the necessity and probable results, and, in doubtful cases, without the sanction of skilful and conscientious counsel, has failed in his duty and dishonored his profession. The author concludes that in nearly all cases supposed to be operable carcinoma uteri we should try to exclude cancer by the microscope, but if this examination confirms the diagnosis of cancer, we should proceed at once to amputation, or, in a certain sense, excision, by the galvano-cautery, and not by vaginal hysterectomy. Abdominal hysterectomy is indicated only in certain cases where the malignant disease is complicated by enlargement, such as by myoma or pregnancy.

Dr. W. B. Gilmer, of Macon, read a paper entitled, "Drainage of the Peritoneal Cavity with the Use of the Syphon Pump."

### THIRD DAY—MORNING SESSION.

Dr. R. M. Harbin, of Calhoun, read a paper entitled, "Trephining in Head Injury with Paralysis of Opposite Arm Followed by Fungous Cerebri," and reported an interesting case which he considered a distinct indication for operative interference. The patient was twenty-five years of age, and in a fight was struck on the head with the heavy end of a thirty ounce billiard cue, and was carried home in an unconcious condition in about two hours thereafter, having vomited a number of times. The patient was seen on the third day after the injury, with the left arm completely paralyzed, sensation being normal. A diagnosis of compression of the brain was made, probably due to a blood clot. Trephining was done on the seventh day after the injury.

Dr. Harbin drew the following deductions from the case:

1. That this patient would almost certainly have died in the first instance had not the clot been removed.

2. That there was a positive indication for trephining.

3. That it furnished a striking example of the practical value of the applied rules of topographical anatomy of the brain.

4. That the prevention of fungous cerebri is more important than the cure, and is not to be looked for without proper precautions.

5. That the occurrence of fungous cerebri is very unusual in such a small trephine wound without injury to the dura mater.

Dr. George H. Noble, of Atlanta, contributed a paper on "Phlegmasia Alba Dolens," and Dr. J. M. Hull, of Augusta, one entitled "Foreign Bodies in the Larynx."

AFTERNOON SESSION.

Dr. Walter A. Crow, of Atlanta, read a paper entitled "Cancer of the Uterus, the Remote Result of Operative Interference," in which he reported a series of cases that had come under his care recently, and which served to impress two facts: (1) The common occurrence of this disease, especially in women approaching the menopause, and (2) the great disposition to overlook their true nature or probable tendency, and to incline to use palliative measures until the disease assumes a malignant form, and we thereby lose our golden opportunity of effecting a cure in most cases.

Dr. H. W. Stafford, of New York, read a paper by invitation on "The Extraction of Clear Lenses for Myopia," and reported five interesting cases. Previous to describing the operations, he related the circumstances which led him to believe that the extraction of the lens, whether clear or not, would arrest the progress of myopia.

"Responsibility and Relation of the General Practitioner to Gynecology, Preventive and Non-Preventive." This was the title of a paper read by Dr. R. R. Kime, of Atlanta, in which he said to properly prepare for special work in gynecology requires years of practical experience in general medicine and obstetrics. The most successful practitioner is one who seeks to know and eliminate as far as possible the cause of disease. In gynecological work it is not so often a question of eliminating the primal cause of disease as it is to correct the results of antecedent causes. To illustrate:

Pyosalpinx is not a primal cause, but the result of a previous inflammation, simple, infectious, or specific. Pelvic peritonitis in the female is not idiopathic, but the result of diseased conditions elsewhere.

Having cited some of the many instances in which the general practitioner may prevent disease and injury to the female and lessen gynecological work, the author then pointed out a few instances in which his duty is reversed, and for the sake of brevity presented them in the form of *Don'ts.*

Dr. J. G. Earnest, of Atlanta, reported a case of extra-uterine pregnancy in a woman thirty-two years of age, the mother of three children.

Dr. J. C. Avary, of Atlanta, read a paper entitled the "Tampon in Gynecology," and Dr. Bernard Wolfe, of Atlanta, one entitled "A Plea for the Closer Recognition of Dermatology as a Specialty."

The following officers were elected: President, Dr. Willis F. Westmoreland, of Atlanta; First Vice-President, Dr. R. H. Taylor, of Griffin; Second Vice-President, Dr. Wm. B. Tate, of Tate; Treasurer, Dr. E. C. Goodrich, of Augusta; Secretary, Dr. D. H. Howell, of Atlanta.

Dr. J. McFadden Gaston, of Atlanta, introduced the following resolution, which was unanimously adopted:

WHEREAS, The American Medical Association at its last meeting provided for an expression in regard to the code of ethics from the different State associations, to be laid before the forthcoming meeting at San Francisco, it is hereby

*Resolved*, That the Medical Association of Georgia reasserts its adoption of, and conformity to, the code of ethics of the American Medical Association, as heretofore recognized by that body, and authorizes this record of its adherence to the same.

After drafting, introducing and adopting resolutions of thanks, the Association, on motion, adjourned to meet in Savannah on the third Wednesday in April, 1895.

